# PPIE-Informed User-Centred Development and Early Usability Evaluation of a Digital Patient Portal to Support Stroke Survivors’ Self-Management

**DOI:** 10.1007/s41666-026-00253-9

**Published:** 2026-09-17

**Authors:** Zhiqiang Huo, Timothy Neate, David Wyatt, Sophie Rowland-Coomber, Fara Hamidi, Martin Chapman, Yanzhong Wang, Iain J. Marshall, Charles D. A. Wolfe, Vasa Curcin

**Affiliations:** 1 Wolfson Institute of Population Health, Queen Mary University of London, London, United Kingdom; 2 Department of Population Health Sciences, King’s College London, London, United Kingdom; 3 Department of Informatics, King’s College London, London, United Kingdom

**Keywords:** Stroke Survivors, Patient Portal, Self-management Support, Stroke Prevention, Electronic Health Records

## Abstract

Stroke survivors often require ongoing support to manage their health, understand personal health information throughout recovery, and reduce the risk of recurrent stroke. Digital patient portals may support self-management, but many existing portals focus primarily on information provision and offer limited integration of longitudinal stroke-specific data, home monitoring, and patient-facing explanations of electronic health records. This study describes the design, development, and early usability evaluation of a patient and public involvement and engagement (PPIE)-informed, user-centred digital portal for stroke survivors, developed in the context of the South London Stroke Register. The portal was refined iteratively using findings from prior qualitative and PPIE work, multidisciplinary stakeholder feedback, and prototype usability testing. The resulting prototype integrates access to personalised health records, home blood pressure monitoring, stroke recurrence risk information, self-assessment tools, educational resources, and caregiver and healthcare contact management. Usability testing with eight stroke survivors indicated that the prototype was broadly acceptable, perceived as useful, and usable in a supported testing environment. This study demonstrates how user-derived evidence and multidisciplinary feedback can be translated into a functional, stroke register-linked patient portal. Future feasibility evaluation will examine real-world acceptability, implementation feasibility, and the portal’s potential contribution to post-stroke self-management in a larger and more diverse population in South London.

**Supplementary Information:**

The online version contains supplementary material available at https://doi.org/10.1007/s41666-026-00253-9.

## Introduction

Stroke is a leading cause of mortality and disability worldwide. In the UK, over 100,000 people have strokes each year [1]. A UK national stroke audit study revealed that cumulative mortality rates rise to 57% within five years and reach 76% within ten years following a stroke [2]. Recent advancements in acute stroke therapy have significantly enhanced short-term outcomes [3]. However, these improvements may not be sustained if stroke survivors cannot access the follow-up care, rehabilitation support, and health information needed to manage long-term recovery after stroke. In particular, following acute treatment, stroke survivors and their families face numerous challenges in managing long-term conditions, such as depression and frailty [4–7]. Research indicates that effective self-management could enhance the quality of life for stroke survivors [8], thereby improving overall well-being and reducing the burden on healthcare services. In this context, post-stroke self-management refers to the ongoing activities through which stroke survivors, often with support from carers and healthcare professionals, monitor their health, manage modifiable risk factors, understand recovery-related information, and make informed decisions about daily care after stroke.

Effective self-management is essential in addressing these challenges, particularly in reducing the risk of stroke recurrence and supporting long-term recovery. Digital health solutions have been increasingly explored as tools to support self-management by enhancing patient engagement, facilitating access to health information, and enabling the routine monitoring of modifiable risk factors. For stroke survivors, monitoring and controlling cardiovascular risk factors such as blood pressure is a key component of secondary prevention. Evidence from a clinical trial indicates that self-monitoring of blood pressure, with or without telemonitoring, can significantly improve blood pressure control in home settings [9], which can facilitate more effective antihypertensive medication titration and reduce cardiovascular risk. Furthermore, adherence to prescribed treatments [10] is important for disease management and reducing the risk of recurrent vascular events. Given the complexities of managing chronic conditions, many stroke survivors highlight the advantages of self-management strategies that incorporate continuous monitoring, personalised feedback, and communication with healthcare providers [11]. Among these digital approaches, patient portals are particularly relevant because they can provide structured access to personal health information, educational resources, and self-monitoring tools.

A patient portal is generally defined as a secure digital platform that enables patients to access personal health information, manage aspects of their care, and communicate with healthcare providers. Such portals have increasingly been used to support patient self-management by improving access to health information and enabling more active involvement in ongoing care [12]. This is particularly relevant after stroke, when survivors may require sustained support beyond the acute care setting to understand their health information, manage modifiable risk factors, and engage with rehabilitation and secondary prevention activities. Patient portals may support these needs by providing access to personal health records [13], educational resources and community support [14], medication reminders [15], home monitoring of indicators such as blood pressure [9], and rehabilitation support beyond clinical settings [16].

Patient portals are increasingly being implemented across a range of healthcare settings [17]. In stroke care, previous studies have developed digital tools to support particular stages or functions of the recovery pathway. For example, Kjörk et al. developed a participatory-design-based pre-visit tool to help stroke survivors prepare for clinical consultations and improve patient–provider communication [18]. Fritz et al. used a co-design approach to develop a website supporting stroke survivors and caregivers during transitions of care [19]. Fakes et al. evaluated a proactive discharge intervention incorporating the “EnableMe” web-based portal to support survivors after hospital discharge [20]. While these studies demonstrate the potential value of digital support across the post-stroke care pathway, existing approaches have typically focused on specific stages or functions of care. Evidence remains limited on how to integrate longitudinal stroke-specific records, home blood pressure monitoring, recurrence risk communication, and patient-facing explanations of health data within a single usable and acceptable portal for stroke survivors.

Developing such an integrated portal is not merely a technical challenge. It also requires careful consideration of how personal health information, monitoring results, and recurrence risk information can be presented in ways that are understandable, accessible, and sensitive to the diverse needs of stroke survivors. Designing effective digital portals for this population remains challenging because users may differ in their health literacy, digital confidence, support needs, and ability to access and interpret information [21, 22]. Portal design must also accommodate the needs of stroke survivors, caregivers, and healthcare providers to ensure usability, engagement, and relevance [23]. This is particularly important because stroke survivors may experience cognitive, linguistic, visual, or other functional impairments that affect their ability to use digital tools [24–26]. For example, stroke survivors with aphasia may find text-heavy interfaces difficult to navigate, while those with visual impairments may require features such as screen reader compatibility and adjustable text sizes [27, 28]. Addressing these challenges requires user-centred and patient and public involvement and engagement (PPIE)-informed approaches to develop portals that are accessible, clinically relevant, and suitable for further evaluation in healthcare settings.

This paper presents the PPIE-informed, user-centred design, development, and early usability evaluation of a digital patient portal for stroke survivors. Developed in South London, the portal responds to a recognised gap in stroke digital health: how longitudinal, stroke-specific follow-up data can be translated into understandable, accessible, and actionable information to support self-management after stroke. The prototype integrates personal health records, home blood pressure monitoring, recurrence risk communication, self-assessment tools, and educational resources within a single patient-facing system. We examine how these functions can be presented in a usable and acceptable form for stroke survivors and report a formative in-person usability evaluation to inform subsequent feasibility testing in a broader and more diverse population.

## Methods

### Study Design and Overview

This study formed part of a wider programme of work developing a digital patient portal to support self-management after stroke [29]. The present study builds on and extends our earlier work [30], which described the initial co-design of the portal and its proposed functions. The current study reports the subsequent translation of user-derived requirements into a functional high-fidelity prototype, iterative refinement informed by multidisciplinary and PPIE feedback, and formative in-person usability testing with stroke survivors.

**Fig. 1:**
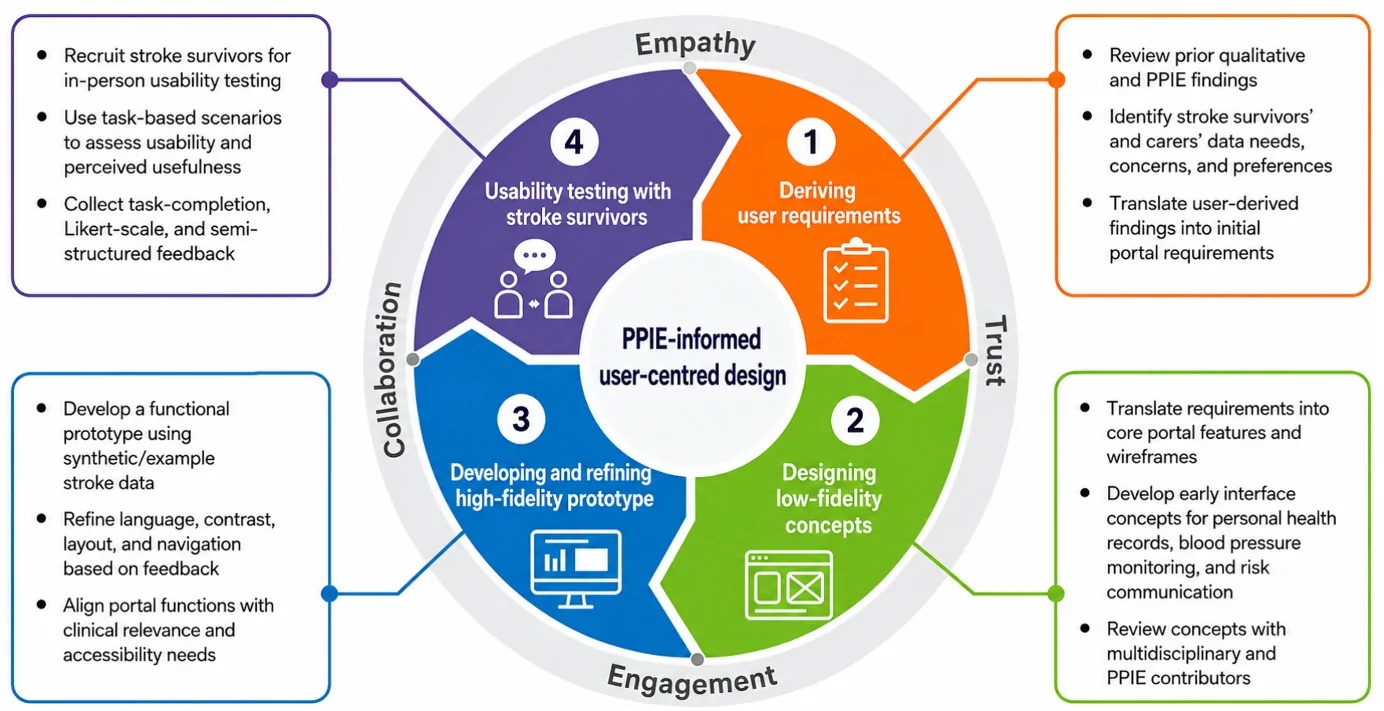
Overview of the PPIE-informed user-centred portal development and usability evaluation process. The process comprised four linked stages: deriving user requirements from prior qualitative and PPIE work, designing low-fidelity portal concepts, developing and refining a high-fidelity prototype through multidisciplinary and PPIE feedback, and conducting in-person usability testing with stroke survivors.

We used a user-centred design approach [31, 32], informed by prior qualitative and PPIE work with stroke survivors and informal carers [33, 34], alongside multidisciplinary stakeholder input from clinicians, researchers, human–computer interaction specialists, and digital health experts. The portal development process followed a four-stage iterative framework, ranging from the identification of user-derived requirements to prototype usability evaluation, as shown in Fig. 1, which was developed by the research team for this study (described in full in Section 2.4).

### Study Setting and South London Stroke Register Context

This study was conducted within the context of the South London Stroke Register (SLSR) [29], a population-based stroke register established in 1995 to monitor the incidence, risk factors, care, and long-term outcomes of stroke in an ethnically diverse inner-city population in South London [35–37]. The SLSR collects clinical, sociodemographic, functional, and follow-up data at multiple time points after stroke, including information relating to acute presentation, hospital care, rehabilitation, and long-term follow-up. This longitudinal data infrastructure provided the clinical and data context for the portal, which was designed to present exemplar stroke-related health information in a patient-facing format.

The user-derived evidence base for portal development was drawn from prior qualitative and PPIE work conducted within the wider programme. This earlier work explored stroke survivors’ and informal carers’ views on accessing personal health data, the perceived benefits and concerns associated with digital health tracking, and the ways in which a patient portal could support post-stroke self-management [33, 34]. The qualitative work included semi-structured interviews with stroke survivors and informal carers and identified issues relevant to portal design. These prior findings were treated as design evidence for the present study. The present study focused on translating these user-derived requirements into a functional prototype and conducting a formative evaluation of its usability and acceptability with stroke survivors.

### Contributors and Participant Involvement

The portal development process involved two main forms of contribution. First, prior qualitative and PPIE work, together with feedback from a multidisciplinary team, informed the early and mid-stage development of the portal. Across the wider development process, 70 participant attendances were recorded in stakeholder and PPIE-related meetings [29]. These figures represent attendances rather than unique individuals, as some contributors participated in more than one activity. The multidisciplinary team included clinicians, stroke researchers, digital health researchers, health informatics researchers, HCI specialists, social scientists, and policy-related contributors.

Different contributors informed different aspects of the portal design. PPIE contributors, including stroke survivors and carers, commented on the clarity, accessibility, language, relevance, and sensitivity of the proposed content. Clinicians and stroke researchers advised on the clinical relevance and interpretation of health information, including blood pressure monitoring and recurrence risk communication. HCI and digital health specialists advised on navigation, accessibility, information structure, and interaction design. The portal development team reviewed the feedback, considered technical feasibility and project scope, and made implementation decisions in consultation with clinical, HCI, and senior project members. Stakeholder and PPIE involvement in the portal development component was consultative and design-informative: contributors shaped requirements and refinements through iterative feedback, while implementation decisions were made by the portal development team.

During the low-fidelity design stage, prototype concepts were discussed in two engagement meetings: one stakeholder meeting with clinicians, researchers, policymakers, and digital health researchers (*n=15* attendances), and one PPIE meeting involving stroke survivors, carers, and researchers (*n=16* attendances). The low-fidelity portal concepts and prototype screenshots were used as discussion tools to gather feedback on the portal’s functions and user experience. During the high-fidelity development stage, the prototype was further reviewed in a stakeholder meeting (*n=15* attendances) and presented through a video demonstration during a PPIE meeting (*n=16* attendances). These discussions informed refinements to the portal’s functions, accessibility features, wording, navigation, and presentation of health information, with the aim of making the prototype more understandable and sensitive to users’ post-stroke experiences.

Second, stroke survivors contributed directly to the formative evaluation of the high-fidelity prototype through in-person usability testing. Eight stroke survivors were invited to use the prototype with a fictional patient scenario and synthetic/example stroke-related data. Feedback from these sessions was used to identify usability issues, clarify user preferences, and inform further refinement of the prototype before feasibility evaluation.

### PPIE-Informed Portal Development and Refinement Process

The portal development process was organised into four linked stages: deriving user requirements, designing low-fidelity concepts, developing and refining a high-fidelity prototype, and conducting usability testing with stroke survivors. The structure was informed by user-centred and participatory design principles in healthcare technology development [31, 38], while the involvement of PPIE contributors and multidisciplinary stakeholders reflected recommendations for maximising patient and public input in digital health design [39]. The four stages were iterative: prior qualitative and PPIE findings served as the evidence base for the initial requirements [33, 34], and feedback from clinicians, researchers, Human-Computer Interaction (HCI) specialists, digital health experts, and PPIE contributors informed subsequent refinements. The methodological literature informing the design of the four-stage process itself, as distinct from this evidence base for the requirements, is discussed separately below.

The four-stage structure was developed by the research team specifically for this project. Its design drew on the broader principles of co-design, in which iterative cycles of requirement-gathering, prototyping, and evaluation are shaped collaboratively with stakeholders positioned along a spectrum of participation [40], and on the internationally recognised human-centred design cycle specified in ISO 9241-210, which describes iterative understanding of context of use, specification of requirements, production of design solutions, and evaluation against those requirements [41]. Embedding PPIE contributors at every stage, rather than only during final evaluation, followed the UK Standards for Public Involvement [42] and recommendations for maximising the value of PPIE within digital health co-design [39]. This staged approach shares a similar overall logic to the three-phase methodology described by Harte et al. for connected health systems [31], which likewise progresses from use-case development, through expert and multidisciplinary prototype review, to user testing with the target population. We extended this structure into four discrete stages by separating low-fidelity concept review from high-fidelity prototype refinement, allowing PPIE and clinical stakeholders to comment iteratively at each level of fidelity; this reflected the added complexity of integrating longitudinal South London Stroke Register data into the portal. The overall pattern also parallels the phased development, multidisciplinary and consumer involvement, and structured usability testing reported by Comachio et al. for a comparable self-management mHealth application [32], although our process was additionally organised around a population-based clinical data infrastructure rather than a single standalone application.

Table 1 summarises how different contributors informed the portal development process and how feedback was translated into design outputs across the four stages.

**Table 1:** Summary of contributor input and design outputs across the portal development and usability evaluation process

Stage	Key contributors	Key activities	Design output or contribution
Deriving user requirements	Qualitative research team, PPIE contributors, and portal development team	Review of prior thematic findings and PPIE evidence relating to stroke survivors’ and carers’ information needs, concerns, and preferences	Defined initial design requirements, including understandable presentation of personal health information, recovery monitoring, sensitive communication of risk-related information, and accessible interface design.
Designing low-fidelity concepts	Portal development team, multidisciplinary stakeholders, and PPIE contributors	Development of Axure wireframes and illustrative user pathways; collection and recording of multidisciplinary and PPIE feedback in a structured feedback-and-action log	Prioritised core portal functions, including personal health record access, blood pressure monitoring, stroke recurrence risk communication, self-assessment tools, and educational resources; identified early design changes for prototype refinement.
Developing and refining high-fidelity prototype	Portal development team, clinicians, human-computer interaction specialists, health informatics researchers, social scientists, senior project team, and PPIE contributors	Development of a functional prototype using synthetic/example stroke-related data; review of logged feedback; agreement of design actions and refinement priorities	Refined wording, navigation structure, layout consistency, colour contrast, information grouping, medication examples, caregiver contact information, and progressive disclosure of detailed content.
Usability testing with stroke survivors	Eight stroke survivors and the portal development team	In-person task-based usability evaluation using a fictional patient scenario and synthetic/example health data; collection of task-completion, Likert-scale, and semi-structured feedback	Assessed usability, acceptability, perceived usefulness, and practical barriers; identified further refinement priorities before feasibility evaluation.

#### Stage 1: Deriving Portal Requirements from Qualitative and PPIE Evidence

The first stage focused on translating prior qualitative findings and PPIE input from SLSR participants within the wider SLSR project into the initial portal requirements. Earlier qualitative work and PPIE activities explored stroke survivors’ and carers’ perspectives on accessing personal health data, managing stroke-related information, understanding recovery-related information, and using digital tools to support self-management after stroke. These prior findings were generated through thematic analysis by the qualitative research team [33, 34].

The portal development team reviewed these user-derived findings and mapped them into initial design domains for the prototype, spanning personal health record access, home blood pressure monitoring, recovery-related information, stroke recurrence risk communication, educational resources, accessibility, and clarity of language. The resulting functional and experiential requirements are reported in full in Section 3.1. These initial domains informed the selection of core portal functions and guided the subsequent development of low-fidelity concepts.

#### Stage 2: Designing Low-Fidelity Concepts

In the second stage, the portal development team translated the initial requirements into low-fidelity concepts and wireframes using Axure. These early concepts were intentionally simple and exploratory, enabling contributors to review the proposed scope, information structure, and user pathways before the development of a functional prototype. The wireframes illustrated example user journeys, including reviewing personal health records, viewing longitudinal stroke follow-up information, interpreting blood pressure readings, accessing educational resources, and viewing stroke recurrence risk information.

The low-fidelity concepts were reviewed by the multidisciplinary and PPIE contributors listed in Table 1, who considered the clinical relevance, technical feasibility, accessibility, and information structure of the proposed functions, and who were shown the early concepts and prototype screenshots to support discussion of the portal’s purpose, content, language, navigation, and presentation of personal health information.

Feedback from these discussions was documented in a structured feedback-and-action log. Each item recorded the issue raised, the source of feedback, the suggested change, and the proposed design action. The portal development team reviewed the logged feedback and considered each item in relation to clinical relevance, accessibility, technical feasibility, and the intended scope of the prototype. Where feedback required further consideration, it was escalated as described in Section 2.3. Actions were recorded as implemented, modified, deferred, or not taken forward, with deferred items retained for consideration during subsequent prototype development or feasibility evaluation. The output of this stage was a prioritised set of design actions to inform the development of the high-fidelity prototype.

#### Stage 3: Developing and Refining the High-Fidelity Prototype

In the third stage, the low-fidelity concepts and prioritised design actions were translated into a functional high-fidelity prototype. The prototype was implemented using the Java Spring Boot 2 framework and was designed to support realistic interaction with the main portal functions [30]. To protect patient privacy and avoid the use of identifiable health information during development, demonstration, and testing, the prototype used synthetic/example stroke-related data, including exemplar demographic information, follow-up records, blood pressure readings, and recovery-related measures [43]. A team-reviewed fictional patient profile was used to demonstrate how personal health information and longitudinal stroke-related data could be presented within the portal.

Before usability testing, the high-fidelity prototype was reviewed through multidisciplinary stakeholder discussions and PPIE activities involving the contributors listed in Table 1. Prototype screenshots, video walkthroughs, and the fictional patient scenario were used to support structured discussion without exposing identifiable patient information. Feedback was sought on clinical relevance, accessibility, navigation, language clarity, presentation of personal health data, recurrence risk communication, and technical feasibility.

Feedback generated during this stage was added to the structured feedback-and-action log and mapped to the design domains described in Section 2.6. The portal development team reviewed the logged feedback and proposed design actions, consulting clinical, HCI, and senior project members where further judgement or scope decisions were required. Priority actions considered necessary for the formative usability evaluation were implemented before the prototype was tested with stroke survivors. Items requiring further technical development, additional clinical review, or consideration beyond the scope of the prototype were retained for future development and feasibility evaluation.

#### Stage 4: Usability Testing with Stroke Survivors

The final stage involved formative in-person usability testing with a pragmatic sample of eight stroke survivors. Participants were recruited from individuals who had previously contributed to PPIE activities within the SLSR programme. Their prior involvement meant that they were familiar with the wider aims of the portal development programme and with the research setting, facilitating attendance at the hospital-based testing sessions. The sample size was selected within the available study timeframe to support an early-stage evaluation of the prototype.

Eligible participants were adults with a history of stroke who were able to provide informed consent, attend an in-person testing session independently or with support from a carer, interact with the tablet sufficiently to complete the supported testing activities, and understand the testing instructions and communicate their responses to the researcher, with support where required. Participants were excluded if they were unable to interact with the provided tablet during the supported session, or unable to understand the testing instructions and engage with the researcher. Cognitive or communication difficulties were not treated as automatic exclusion criteria. Two of the eight participants required some additional support during the session but were able to provide informed consent and engage with the testing activities.

Participants were not required to own or bring a personal digital device, as all necessary equipment was provided by the research team. Prior experience of using digital health platforms was not required. Before testing began, the researcher explained the purpose of the study, the main functions of the portal, and the testing procedure. Each participant completed the usability session individually, with a researcher present to provide guidance and light, non-directive support where required. Participants received compensation in accordance with institutional guidance. The sessions were conducted at Guy’s Hospital in South London and lasted approximately 40 minutes per participant.

Participants interacted with the high-fidelity prototype using the fictional patient scenario and synthetic/example stroke-related data described in Section 2.4.3, enabling completion of realistic tasks without the use of identifiable health information.

Each session included four components. First, an initial impression task captured participants’ first responses to the portal. Second, participants completed nine structured interaction tasks covering key portal functions, including locating personal health records, interpreting blood pressure information, reviewing stroke recurrence risk information, and navigating educational content. Third, participants completed a 5-point Likert-scale questionnaire assessing perceived usability, acceptability, usefulness, and confidence in using the portal. This questionnaire was developed in-house by the research team for this study; item selection and wording were reviewed by human–computer interaction researchers and social scientists within the team to ensure that questions were interpretable and used plain, accessible language appropriate for stroke survivors. Finally, semi-structured feedback was collected to explore views on content clarity, navigation, accessibility, risk communication, device preferences, training needs, and suggestions for further refinement. The usability testing was conducted as a formative prototype evaluation to inform further refinement before feasibility testing.

### Data Collection

Data collection comprised three sources corresponding to the portal development and usability evaluation components of the study. First, prior qualitative and PPIE findings generated within the wider programme were used as pre-existing design evidence to inform the initial portal requirements (see Section 2.2).

Second, during the low-fidelity and high-fidelity prototype development stages, feedback was collected from multidisciplinary stakeholders and PPIE contributors through review meetings, prototype demonstrations, and structured discussions. Data sources included meeting notes, written comments, researcher field notes, and a structured feedback-and-action log. These records captured the feedback source, issue raised, suggested refinement, proposed design action, and implementation status.

Third, usability evaluation data were collected during in-person testing sessions with eight stroke survivors using the fictional patient scenario and synthetic/example stroke-related data described in Section 2.4.3. The usability dataset included task-completion records for nine structured interaction tasks, responses to a 5-point Likert-scale questionnaire assessing usability, acceptability, perceived usefulness, and confidence, and brief semi-structured feedback on participants’ experiences of using the prototype. These data were used to assess whether the portal was understandable, acceptable, and practically usable in a supported testing context, and to identify priorities for further refinement before feasibility evaluation.

### Design Mapping and Usability Data Analysis

Analysis was structured around two components: design mapping of development-stage feedback and analysis of usability evaluation data. Prior qualitative and PPIE findings were treated as pre-existing evidence rather than reanalysed as a new qualitative dataset (see Section 2.2) [33, 34].

Development-stage feedback from stakeholder meetings, PPIE activities, and prototype demonstrations was analysed using a framework-based design mapping approach. Feedback records were reviewed and mapped deductively to predefined design domains, including content and language, accessibility and navigation, clinical relevance, emotional sensitivity of risk-related information, presentation of personal health data, technical feasibility, and implementation readiness. The purpose of this mapping was to maintain an audit trail between user or stakeholder input and subsequent prototype refinements, rather than to generate new qualitative themes. Mapped feedback items were summarised narratively and used to identify illustrative examples of how design requirements were translated into prototype features and refinements.

The design mapping process was led by the portal development team and reviewed iteratively throughout prototype development. Feedback items and proposed design actions were initially reviewed by the lead researcher and discussed with relevant members of the multidisciplinary team when interpretation, feasibility, or scope decisions required further consideration. Final design actions and implementation priorities were reviewed collectively to ensure alignment with user needs, clinical relevance, accessibility requirements, and the intended scope of the prototype.

Usability testing data were analysed descriptively. For the nine structured interaction tasks, each task was coded as completed or not completed according to whether the participant completed the task independently or with light, non-directive support [44]. Task-completion rates were calculated for each task and across all participants to identify functions that were readily usable and areas where interaction difficulties occurred.

Likert-scale questionnaire responses were summarised using frequencies across the five response categories. Overall agreement was calculated by combining ‘Agree” and ‘Strongly Agree” responses [45]. No inferential statistical testing was conducted because the usability evaluation was exploratory and involved a small sample of stroke survivors.

Semi-structured feedback from the usability sessions was analysed separately from development-stage feedback to focus specifically on participants’ experiences of using the high-fidelity prototype. Feedback was organised using framework-based content analysis around usability-focused domains, including ease of use and first-time navigation, clarity of personal health information, interpretation of blood pressure trends, interpretation and acceptability of stroke recurrence risk information, accessibility, device preferences, onboarding or training needs, and suggested refinements. These qualitative findings were then compared with task-completion and questionnaire results to contextualise areas of strong performance, explain tasks or questionnaire items where difficulties were observed, and identify priorities for further prototype refinement before feasibility evaluation.

### Ethical Approval

The wider research programme from which the portal development was derived received ethical approval from the Health Research Authority (HRA) and Health and Care Research Wales (HCRW) Research Ethics Committee (REC Reference: 21/WS/0145). The usability evaluation reported in this study received additional ethical approval from the Research Ethics Office at King’s College London (Reference: LRS/DP-23/24-41635). All participants involved in the usability testing provided written informed consent prior to participation.

## Results

The results are presented according to how prior qualitative and PPIE findings, stakeholder and PPIE feedback collected during portal development, and usability testing informed the development and refinement of the patient portal. We first summarise how user-derived evidence and development-stage feedback were translated into design actions, followed by the resulting prototype features and usability testing findings. Table 2 provides illustrative examples of how stakeholder and PPIE feedback was mapped to design actions during prototype development. A more detailed feedback-to-design mapping table is provided in Supplementary Material 1.

**Table 2:** Examples of feedback-to-design mapping during portal development

Design domain	Feedback source	Issue raised	Design action
Content and language	Stakeholder	Stroke type and medication terms may be difficult to understand without further explanation.	Added plain-language descriptions and links to trusted educational resources.
Personal data presentation	Stakeholder & PPIE feedback	Mock-up demographic data could create assumptions around gender, name, or title.	Added salutation/profile fields and reviewed fictional patient details used in the prototype.
Medication examples	Stakeholder feedback	Medication examples should align with the example stroke type, particularly for haemorrhagic stroke.	Removed inappropriate blood-thinning medication from the demonstration scenario and reviewed medication examples.
Emotional sensitivity	Stakeholder feedback	Recovery scores and population comparison may be discouraging for some users.	Removed or softened comparative/population score presentation and used more supportive explanatory wording.
Accessibility	Prior qualitative & PPIE findings	Some users may struggle with reading, typing, or communication difficulties such as aphasia.	Included text-to-speech support and considered alternative input or support options for future development.
Carer involvement	PPIE feedback	Family and caregiver support should be visible after discharge.	Added family and caregiver contact information within the portal.
Blood pressure monitoring	Stakeholder feedback	The portal should clarify whether and how users can log blood pressure readings and use them to support recurrence prevention.	Developed blood pressure logging and visualisation functions with explanatory context.

### User-Derived Requirements and Low-Fidelity Design Priorities

Portal requirements were derived from two linked evidence sources: prior qualitative and PPIE findings from the wider programme, and feedback collected during stakeholder and PPIE activities within the portal development component. Together, these sources informed two overarching categories of requirements. Functional requirements referred to the portal features needed to support post-stroke self-management, while experiential and accessibility requirements referred to how personal health information should be presented, explained, and navigated.

Four functional requirements were identified as central to the prototype. First, the portal needed to provide structured access to personalised stroke-related health information, including stroke onset details, follow-up records, medication-related information, and longitudinal recovery measures. Second, it needed to support home-based health monitoring, particularly blood pressure recording, trend visualisation, and interpretation. Third, it needed to include self-assessment tools that could help stroke survivors understand changes in recovery, function, and quality of life over time [46]. Fourth, it needed to provide centralised access to trusted educational and support resources.

Across these functional areas, contributors emphasised several experiential and accessibility requirements. Information needed to be presented in plain and patient-friendly language, with clinical terms explained rather than simply displayed. Personal health data needed to be labelled clearly so that users could understand what type of information was being shown and why it was relevant to self-management. Recovery trajectories, clinical scores, and stroke recurrence risk information needed to be presented sensitively, with supportive explanations and without unnecessarily alarming visual cues. Contributors also highlighted the importance of reducing cognitive load through simple navigation, consistent layouts, clear visual hierarchy, strong contrast, and flexible access to additional information.

Mapping these requirements into the low-fidelity design stage produced three design priorities that shaped the subsequent prototype. The first priority was structured access to personal stroke-related information, allowing users to view key elements of their stroke history and follow-up information in an understandable format. The second priority was home blood pressure monitoring, including recording, trend visualisation, and plain-language interpretation to make monitoring data practically useful for self-management and clinical discussion. The third priority was sensitive communication of stroke recurrence risk, including presentation of both current estimated risk and potential improvement associated with modifiable factors such as better blood pressure control.

These priorities informed the scope, content structure, and interaction flow of the high-fidelity prototype. They also guided the refinement of wording, information grouping, navigation, accessibility features, and progressive disclosure of more detailed content. A detailed summary of the user-derived functional, experiential, and accessibility requirements, together with their corresponding design implications for the portal, is provided in Supplementary Material 2.

### High-Fidelity Prototype Functions and Design Refinements

**Fig. 2:**
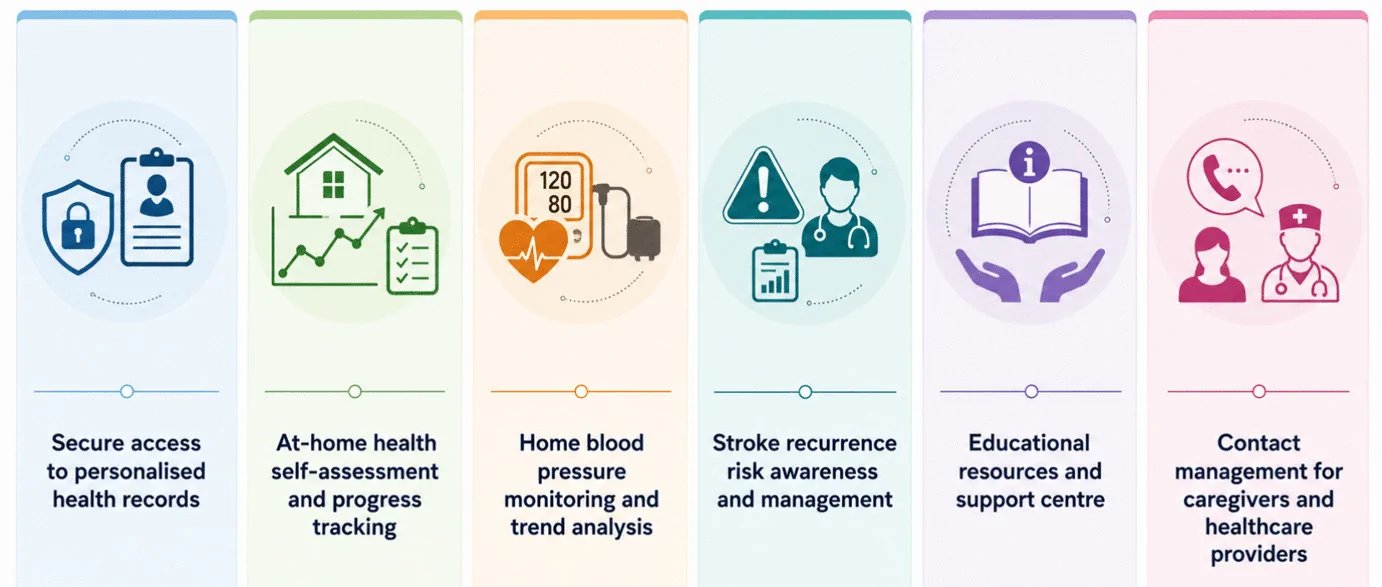
Core functional components of the high-fidelity patient portal prototype

Following the low-fidelity design stage, the prioritised requirements and feedback recorded in the feedback-and-action log were translated into a functional high-fidelity prototype. The resulting prototype integrated core functions intended to support stroke survivors’ access to personal health information, self-monitoring, risk understanding, and access to trusted support resources. The main functional components are summarised in Fig. 2.

The high-fidelity prototype included six core functional areas. First, it provided access to personalised stroke-related health records, allowing users to view exemplar long-term follow-up information derived from the SLSR context. Second, it included a self-assessment area incorporating measures such as the Barthel Index and EQ-5D-5L to illustrate how recovery-related information could be presented over time. Third, it included a blood pressure monitoring function with step-by-step guidance, recording functions, visualisation of trends, and plain-language interpretation of readings. Fourth, it included a stroke recurrence risk module designed to support understanding of individual risk information and the potential relevance of modifiable factors. Fifth, it provided a curated educational resource area to support stroke literacy and self-management. Sixth, it included contact information for family members or caregivers to reflect feedback on the importance of support networks in post-stroke care.

The prototype was also refined to improve accessibility, clarity, and ease of navigation. Feedback from HCI and digital health contributors informed the design of an overview landing page, a shallow two-layer navigation structure, grouped information panels, and expandable “More” sections to reduce cognitive load while allowing users to access additional detail when needed. Visual accessibility was addressed through improved colour contrast and layout consistency, with contrast checked using the WAVE accessibility evaluation tool. The prototype also incorporated multiple information formats, including text-to-speech support, to accommodate different user preferences and accessibility needs.

**Fig. 3:**
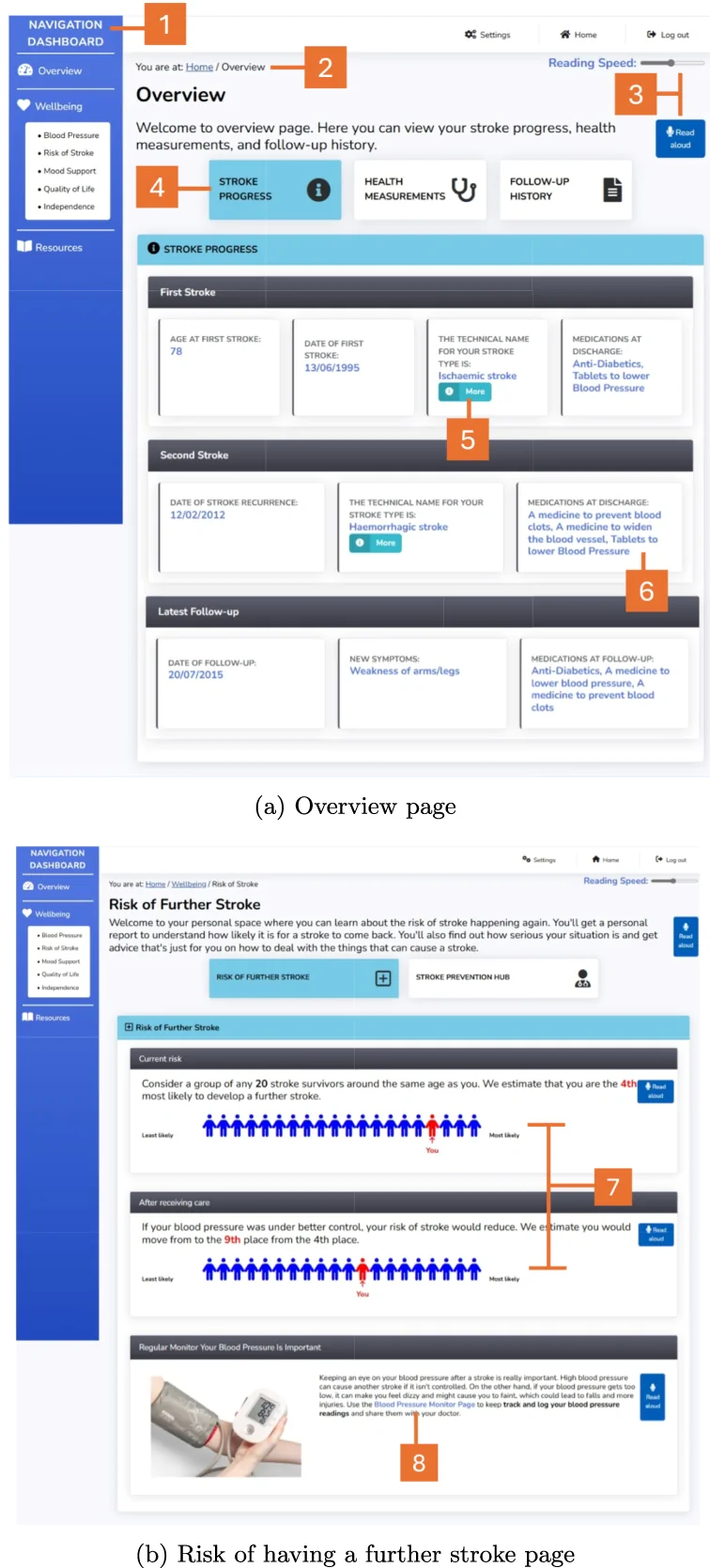
Snapshots of the user interface: overview page and stroke risk page with key UX features. This figure displays selected interface screens highlighting user-centred design features. Notable functions include: (1) a shallow navigation dashboard allowing access up to two levels deep for ease of use; (2) a structured breadcrumb trail to indicate user location; (3) a text-to-speech function with adjustable playback speed for each content section; (4) thematically grouped information panels to toggle between high-priority topics; (5) expandable sections offering additional detailed content on demand; (6) use of accessible, user-friendly language to explain clinical terms such as medications; (7) personalised risk indicators presented through illustrative graphics, showing both the current estimated risk and potential improvement after intervention, accompanied by appropriate guidance on risk management; and (8) explanations of the benefits of effective blood pressure management in reducing the risk of recurrent stroke

Several refinements were made to the presentation of personal health data. Clinical terms and medication-related information were revised using plain-language explanations, and risk-related information was presented with supportive wording and contextual explanations. Information was grouped by topic to help users understand where they were within the portal and why each section was relevant. Illustrative screenshots of the overview page and stroke recurrence risk page are shown in Fig. 3.

### Usability Testing Findings

**Fig. 4:**
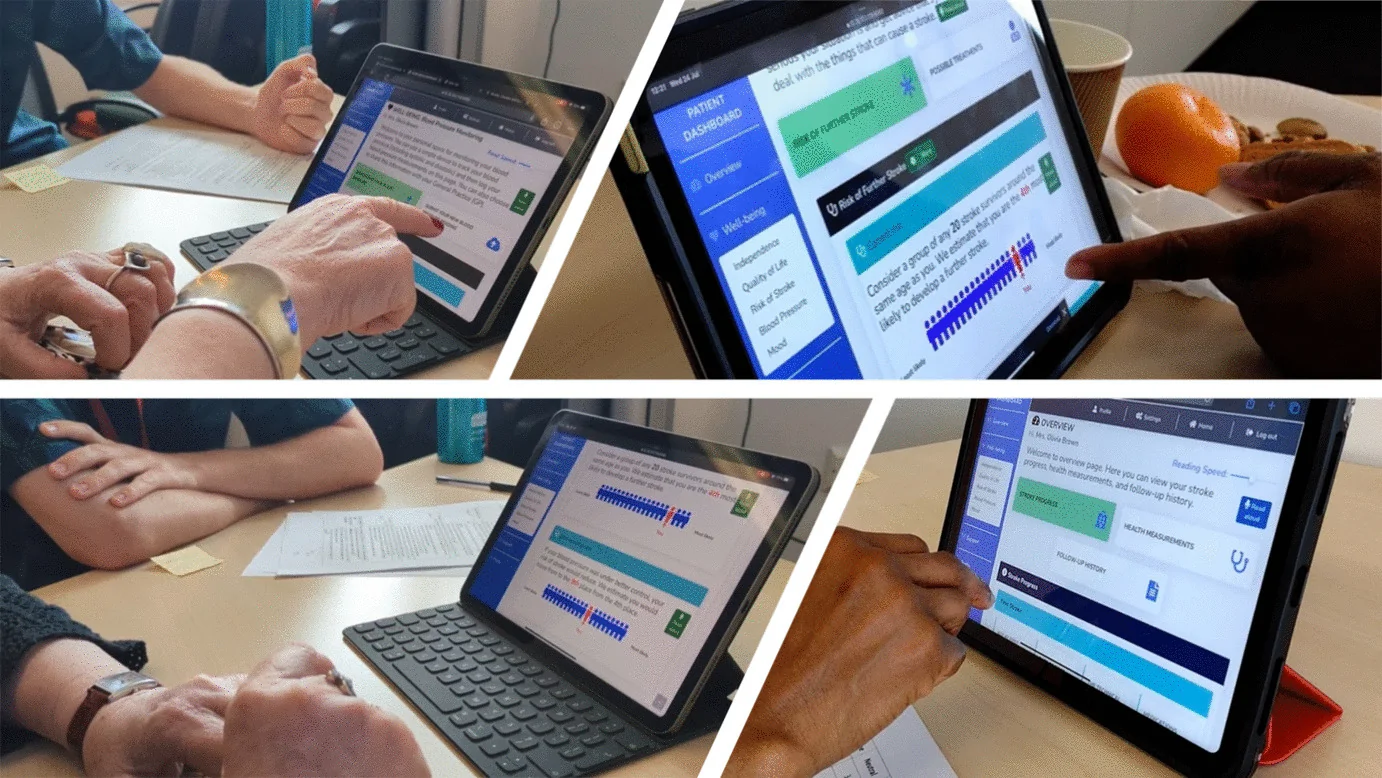
An example photo of a participant using the portal to view personal stroke health information and progress with 10-year follow-up example data (synthetic data examined by the research team)

Usability testing was used to examine whether the PPIE-informed high-fidelity prototype was understandable, acceptable, and practically usable for stroke survivors. Eight stroke survivors took part in the in-person usability testing session (mean age = 69.8 years, 95% CI [63.9, 75.6]; 62.5% female). Participant characteristics are shown in Table 3. Figure 4 shows an example of a participant interacting with the prototype during the testing session. Participants completed an initial impression task, nine structured interaction tasks, a 5-point Likert-scale questionnaire, and semi-structured feedback.

**Table 3:** Stroke survivors participating in the study

ID	Gender	Age	Stroke Severity
Patient 1	Female	72	Mild
Patient 2	Female	64	Moderate
Patient 3	Female	60	Moderate
Patient 4	Male	83	Mild
Patient 5	Female	72	Mild
Patient 6	Female	55	Mild
Patient 7	Male	75	Moderate
Patient 8	Male	77	Mild

#### Task Completion

As shown in Table 4, participants completed a mean of 8.3 out of 9 tasks, corresponding to an overall task completion rate of 93%. Tasks involving interpretation of blood pressure trends, understanding stroke recurrence risk information, and reviewing risk-related recommendations (T5, T7–T9) were completed by all participants. Tasks involving multi-step navigation or locating specific information, such as identifying medication information, finding blood pressure records, or logging a new blood pressure reading (T1–T4, T6), had a completion rate of 87.5%.

**Table 4:**
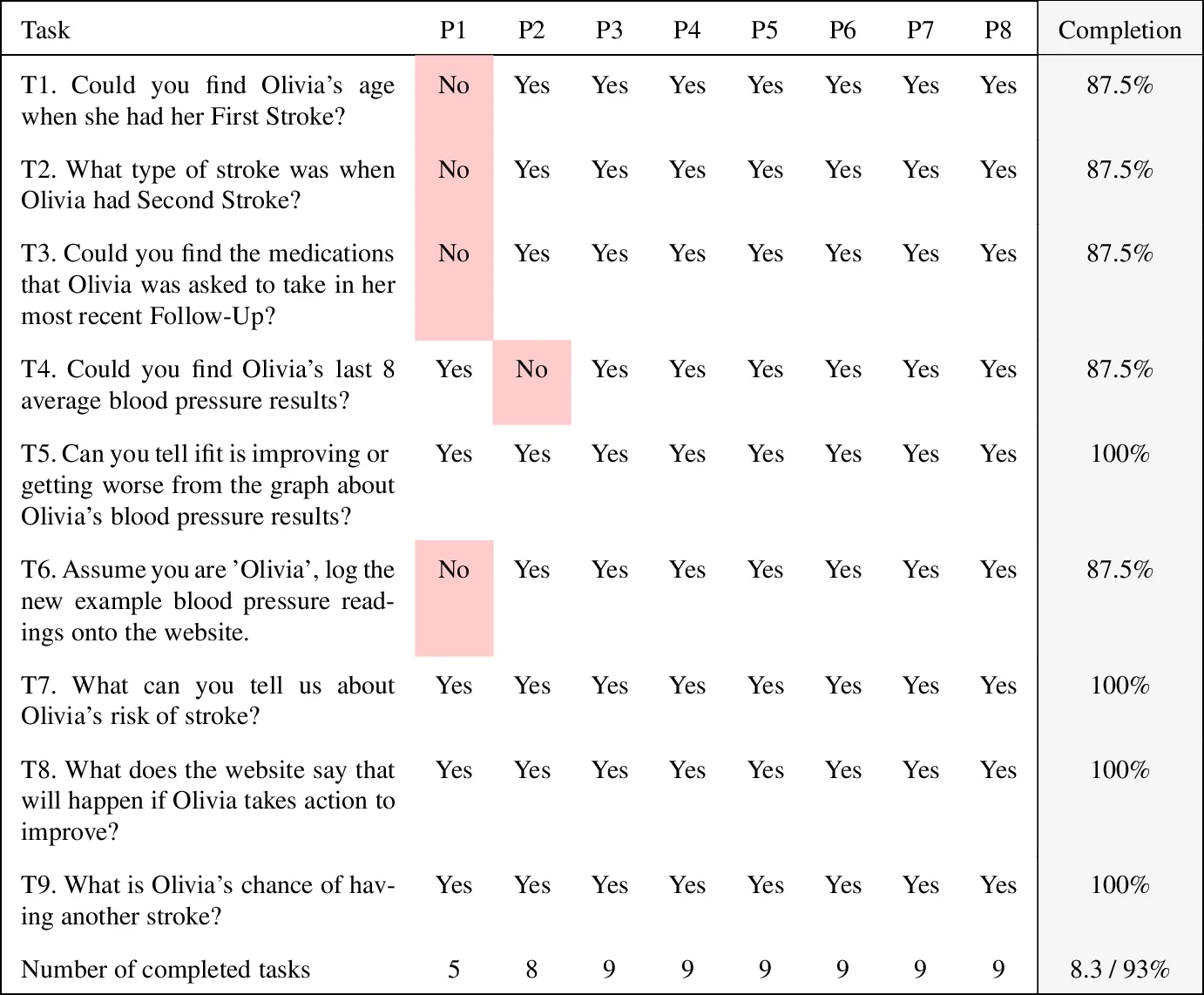
Task completion rates by participants during the usability study using ‘Olivia’ as the example stroke survivor

One participant did not complete several tasks during the session. The remaining participants completed most tasks independently or with light, non-directive support. These findings suggest that the core portal functions were generally understandable in a supported testing context, while also identifying areas where navigation cues, task guidance, and explanation of visual information could be improved before feasibility evaluation. Difficulties were concentrated in information-locating and data-entry tasks rather than in interpreting the overall meaning of blood pressure trends or recurrence risk information, suggesting that the main usability barriers related to navigation cues, interaction guidance, and first-time use rather than the perceived relevance of the portal content.

#### Questionnaire Responses

Questionnaire responses indicated generally positive perceptions of the prototype’s usability, acceptability, and usefulness. As shown in Table 5, 63 of 72 item-level responses (87.5%) were rated as either ‘Agree” or ‘Strongly Agree”. All eight participants agreed or strongly agreed that personalised information about the risk of having another stroke would be useful (Q5) and that they would use the website to support post-stroke self-management (Q7). Responses were also generally positive regarding the relevance of the information presented on the website (Q8), with seven of eight participants selecting ‘Agree” or ‘Strongly Agree”.

Some variation was observed across questionnaire items. Responses to Q1 (ease of use) and Q3 (whether the website design helped users understand information) included a small number of negative or neutral ratings, suggesting that aspects of layout clarity, first-time navigation, or explanation of interface elements may require further refinement. Similarly, Q9 (whether the website contained all expected functions and features) indicated that expectations around feature completeness may vary among users. These findings were treated as formative feedback for improving the prototype rather than as evidence of effectiveness. These questionnaire findings were therefore interpreted alongside the semi-structured feedback to understand why participants perceived the portal as useful overall, while still identifying specific needs for clearer visual explanation, onboarding support, and refinement of first-time navigation.

**Table 5:** Likert responses to user experience and evaluating the usability of high-fidelity patient portal prototype

Question	Strongly Disagree	Disagree	Neutral	Agree	Strongly Agree
Q1. The website is easy to use.	1	1	0	5	1
Q2. The user interface of this website is pleasant.	0	0	1	5	2
Q3. The website design helped me easily understand information on the website.	0	1	1	2	4
Q4. The website helped me easily understand blood pressure readings.	0	0	1	2	5
Q5. The personalised prediction of the risk of having another stroke is useful.	0	0	0	3	5
Q6. I feel comfortable using this website.	0	0	1	5	2
Q7. I would use this website to support my stroke rehabilitation.	0	0	0	3	5
Q8. The information on the website is relevant.	0	0	1	5	2
Q9. This website contains all the necessary functions and features that I expect it to have.	0	0	1	5	2
Total	**1**	**2**	**6**	**35**	**28**

#### Qualitative Usability Feedback

Semi-structured feedback provided further insight into participants’ experiences of using the high-fidelity prototype and helped contextualise the task-completion and questionnaire findings. Feedback was organised around five usability-focused themes: overall acceptability and perceived usefulness, understanding of personal health records and blood pressure information, interpretation of stroke recurrence risk information, navigation and digital confidence, and preferences for future implementation.

##### Overall Acceptability and Perceived Usefulness

Participants generally responded positively to the portal and viewed it as a potentially useful tool for supporting post-stroke self-management. Feedback suggested that the clear layout, plain-language explanations, and structured presentation of stroke-related information helped participants understand the purpose of the portal. This was consistent with the questionnaire findings, where most responses were positive and all participants agreed or strongly agreed that they would use the website to support their post-stroke rehabilitation. Participants particularly valued the idea of bringing together personal health information, monitoring tools, and educational resources in one place, rather than needing to access information across separate systems or paper-based records.

##### Understanding Personal Health Records and Blood Pressure Information

Participants found the personal health record and blood pressure monitoring functions relevant to their everyday self-management needs. The ability to view stroke-related information, record blood pressure readings, and track changes over time was viewed as helpful for understanding recovery and supporting future discussions with healthcare professionals. For example, one participant described the value of stored blood pressure records and average readings: “*Yes, I have a blood pressure monitor and it’s very good. I think it holds up to 50, whatever you call them, records, and it tells you the average at that moment in time, which is very helpful.*” This aligned with the task-completion results, where all participants were able to interpret whether the example blood pressure graph was improving or worsening. However, tasks that required locating specific information or entering new blood pressure readings showed slightly lower completion rates, suggesting that users may benefit from clearer prompts, more visible navigation cues, and step-by-step guidance for data-entry tasks.

##### Risk Communication was Valued But Required Guided Explanation

The stroke recurrence risk module was generally viewed as acceptable and useful, particularly because risk information was presented with explanatory text and linked to potentially modifiable factors such as blood pressure control. This was consistent with the questionnaire findings, where all participants agreed or strongly agreed that personalised information about the risk of having another stroke would be useful. The task-completion findings also suggested that participants could understand the main message of the risk page in a supported testing context, as all participants completed the risk-related tasks. Nevertheless, some participants found the visual risk indicators difficult to interpret without additional explanation. This indicated that future versions should provide clearer labels, more explicit interpretation of risk graphics, and guided explanations to help users understand what the visual information means and how it relates to self-management actions.

##### Navigation, Onboarding, and Digital Confidence Shaped Usability

Although participants generally responded positively to the shallow navigation structure and layered presentation of information, feedback also indicated that first-time use could be challenging for some users. Several participants had limited prior experience with digital health platforms, and some needed orientation to understand where information was located and how to move between sections. This finding was consistent with the small number of negative or neutral questionnaire responses for ease of use and whether the design helped users understand information. Participants’ feedback suggested that brief onboarding, introductory guidance, or a demonstration mode may be needed before routine independent use, particularly for stroke survivors with lower digital confidence or additional cognitive, visual, or communication support needs.

##### Device Preferences and Future Implementation Needs

Participants highlighted the importance of flexible access across devices, including tablets and smartphones. Feedback suggested that the portal should be usable in different home contexts and should accommodate varying levels of digital confidence and support from family members or carers. Participants also supported layered content presentation, such as expandable “show more” sections, because this allowed users to access additional information progressively without being presented with too much detail at once. These findings informed refinement priorities for the next stage of feasibility evaluation, including clearer visual explanations, improved onboarding support, device optimisation, further testing of navigation and information hierarchy, and exploration of how the portal could be used independently or with carer support in real-world settings.

## Discussion

### Principal Findings

This study reports the development and early usability evaluation of a PPIE-informed, user-centred digital portal designed to support self-management after stroke. The portal development process translated prior qualitative and PPIE findings into a functional high-fidelity prototype and refined it through multidisciplinary and PPIE feedback. Usability testing with eight stroke survivors provided preliminary evidence of acceptability and perceived usefulness, while also identifying areas requiring further refinement before feasibility evaluation.

A key finding from the development process was that stroke survivors and stakeholders valued access to personal health information when it was presented in a structured, understandable, and supportive format. Stroke survivors emphasised the importance of accessing personalised health progress and follow-up records, while clinicians highlighted the need to present health-related data in ways that are clinically meaningful and appropriate for self-management. For this reason, acute stroke symptoms—such as sudden changes in vision, speech, or motor function—were not prioritised as retrospective feedback items within the portal, as these are typically recorded at stroke onset and are less suited to ongoing self-management feedback. In contrast, home-based blood pressure monitoring was identified as a more actionable and relevant long-term self-management function. This aligns with evidence that self-monitoring of blood pressure can support better blood pressure management in home settings [9]. In the prototype, this informed the inclusion of functions allowing users to record, view, and interpret self-monitored blood pressure readings.

Another important design output was the inclusion of stroke recurrence risk information. Stakeholders highlighted the potential value of communicating recurrence risk to support awareness of modifiable factors. The usability testing findings suggested that participants perceived personalised stroke recurrence risk information as useful, particularly in the Likert-scale item on the usefulness of personalised stroke risk prediction (Table 5).

Educational resources were incorporated in response to user preferences for centralised access to reliable stroke-related information and support. This is consistent with previous work highlighting the importance of accessible patient education and support resources for people living with cardiovascular conditions [47, 48]. Participants also highlighted the importance of accessing localised stroke recovery resources, which has similarly been noted in previous work on stroke transition support [19]. In the present prototype, educational content and signposting were therefore included to support users in finding relevant information without needing to search across multiple external sources.

User experience and accessibility were central to the design process, particularly because many stroke survivors are older adults, may have limited digital or health literacy, and may experience cognitive, language, or communication impairments [27]. The resulting design choices, including layered information formats and plain-language revision of clinical terminology, are summarised in Section 3.2.

### Interpretation of Design Decisions

The development process involved several design decisions where clinical relevance, user understanding, emotional sensitivity, and technical feasibility needed to be balanced. One example was how to present risk-related information. Some stakeholder feedback supported the inclusion of pre-stroke risk factors to raise awareness, while other feedback suggested that this could be confusing, incomplete, or discouraging if not presented with sufficient context. In response, the development team refined the risk-related content to focus on information that could be interpreted meaningfully by users and linked to potential self-management actions.

The term “risk factors” was also considered potentially unclear or intimidating for some users. This led to a greater emphasis on patient-friendly language and actionable explanation. Similarly, the presentation of “stroke recurrence” generated mixed but constructive feedback. Some contributors suggested alternative wording such as “second stroke” or “third stroke”, while others raised concerns about the emotional impact of recurrence-related language. The decision to retain stroke recurrence risk information reflected its clinical relevance, but the presentation was refined to use supportive wording, contextual explanation, and progressive disclosure. A stroke recurrence risk dashboard developed in previous work by the wider research team [49] informed the conceptual basis for presenting recurrence risk in a patient-facing format. In the present study, usability testing provided early feedback on the clarity and perceived usefulness of this feature, but further feasibility testing is needed to assess how users interpret and act on this information in routine care.

These design decisions highlight the importance of treating personal health data as information that requires interpretation, explanation, and careful framing. Presenting data alone is unlikely to be sufficient. For stroke survivors, information about recovery, blood pressure, risk, medication, or follow-up history may support self-management only when it is understandable, relevant, and emotionally appropriate. The feedback-to-design mapping process used in this study helped translate stakeholder and PPIE input into concrete refinements, including changes to wording, navigation, layout, colour contrast, information grouping, and the use of expandable content.

### Comparison with Prior Work

This study contributes to the growing literature on digital tools and patient portals designed to support self-management after stroke and other long-term conditions. Several previous studies have developed co-designed, participatory, or user-informed digital systems for stroke care, rehabilitation, or chronic disease management. However, many existing tools focus on narrower aspects of care, such as transitional support, rehabilitation, education, or general self-management, and fewer studies integrate longitudinal stroke-specific health records, home blood pressure monitoring, stroke recurrence risk communication, and structured usability testing within a single patient-facing prototype.

Table 6 compares the key features of our portal with six recent studies in stroke or cardiovascular care. Previous systems, such as those by Fritz et al. [19], Kjörk et al. [18], and Song et al. [50], provide important examples of participatory or user-centred digital health development. However, these studies generally do not combine personalised stroke health record access, home blood pressure monitoring, and recurrence risk communication in one platform. Other studies, such as Silvera et al. [53] and Gibson et al. [51], include secondary prevention features, but do not specifically integrate longitudinal stroke-register data or individualised stroke recurrence risk information.

**Table 6:** PPIE-informed user-centred design, development, and early usability evaluation of a stroke register-linked patient portal to support post-stroke self-management.

Study	Aim	Personal Health Records Access	Home Blood Pressure Monitoring Support	Stroke Recurrence Risk	Educational Resources	Health-related Self-assessment	Target Users	Usability Testing
[19], 2021	Develop a patient-centred website to support stroke patients and caregivers during care transitions	No	No	No	Yes	No	Stroke survivors and caregivers	No
[50], 2021	Design mHealth app for chronic disease management using clinician-led co-design	No	No	No	Yes	Yes	Patients with chronic conditions (e.g., obesity)	No
[18], 2022	Develop a digital previsit tool for stroke survivors using participatory design	No	No	No	No	Yes	Stroke survivors	No
[51], 2024	Develop a digital health intervention for CVD secondary prevention with end-user input	No	No	No	Yes	Yes	General patients with CVD	Yes (n=9)
[52], 2023	Design a digital system to support stroke rehabilitation	No	Yes	No	Yes	Yes	Stroke survivors	No
[53], 2024	Co-design a digital support program for stroke/TIA secondary prevention	No	Yes	No	Yes	Yes	Stroke and TIA survivors	Yes (n=4)
This study	Develop and evaluate a PPIE-informed portal to support post-stroke self-management	Yes	Yes	Yes	Yes	Yes	Stroke survivors	Yes (n=8)

Abbreviations: CVD – Cardiovascular Disease; TIA – Transient Ischemic Attack. In this study, personal health records refer to data archived at hospital admission or during clinical follow-ups. Health-related self-assessment refers to functionalities that help users track their health progress, which may include clinically recognised assessments

The present portal extends this body of work by bringing together multiple self-management functions in a single prototype. It enables access to personal health records archived at hospital admission or follow-up, incorporates stroke-specific home blood pressure monitoring, presents stroke recurrence risk information, and includes health-related self-assessment tools such as the Barthel Index and EQ-5D-5L. The study also adds early usability evidence from stroke survivors, with a task completion rate of 93% and generally positive questionnaire responses for perceived usefulness and relevance. These findings support the suitability of the prototype for further feasibility evaluation.

A further strength of this work is its grounding within the SLSR, a population-based cohort with over 20 years of longitudinal stroke data [37]. This context enabled the prototype to be designed around stroke-specific variables and follow-up information that are relevant to long-term care. Rather than claiming broad generalisability at this stage, the SLSR context supports the clinical and contextual relevance of the prototype and provides a strong basis for future feasibility testing in a real-world stroke follow-up setting.

### Design and Implementation Implications

This study provides several lessons for the design and future implementation of digital health tools for stroke survivors, building on the PPIE-informed and multidisciplinary process described above.

As noted above, this required treating personal health data as information requiring interpretation and contextualisation rather than simple display; the feedback-to-design mapping process helped translate this principle into concrete refinements such as plain-language explanations, topic-based grouping, and progressive disclosure.

This balance between information provision and emotional sensitivity, discussed above, is particularly relevant for future digital tools that aim to communicate risk or longitudinal recovery information to people living with long-term consequences of stroke.

Implementation in routine care will require further attention to digital and health literacy, onboarding, and clinical workflow integration. Future deployment would therefore need to consider brief training, supported onboarding, and clear routes for help, alongside integration with primary care or stroke follow-up workflows to avoid creating additional workload for clinicians or unrealistic expectations for patients and carers.

Our experience also reflects wider challenges in developing digital health interventions with end users, including participant recruitment, sustained engagement, training needs, and the need to translate diverse feedback into feasible design actions [54]. Future technical developments may help address some of these challenges. For example, large language models could potentially support personalised, context-sensitive explanations to help users navigate complex interfaces, interpret health data, or complete self-assessments [55]. Similarly, Bluetooth-enabled monitoring technologies could reduce the burden of manual blood pressure entry and support more consistent home monitoring [56]. These technologies should be evaluated carefully in future work to determine whether they improve usability, accessibility, and safe self-management in real-world settings.

### Limitations

Several limitations should be noted. First, the usability testing was conducted with a small group of eight stroke survivors in South London. This sample was appropriate for early-stage formative usability evaluation, but it cannot support conclusions about clinical effectiveness, long-term engagement, or generalisability to wider stroke populations or healthcare systems. Broader testing with a more diverse sample will be needed to understand how the portal is used by stroke survivors with different levels of impairment, digital confidence, language needs, cultural backgrounds, and access to technology. Further, usability participants were recruited from individuals who had previously contributed to PPIE activities within the wider SLSR programme. They may therefore have been more engaged with research activities or more willing to discuss digital health tools than stroke survivors who had not previously participated in PPIE work.

Second, the usability evaluation was conducted in a supported testing environment using a fictional patient scenario and synthetic/example stroke-related data. This approach was important for privacy and safety, and allowed participants to interact with realistic portal functions without exposing identifiable health information. However, it may not fully reflect how users would engage with their own personal health information in everyday settings. Future feasibility work should examine how stroke survivors interpret and respond to their own data over time, and whether additional support is needed when information is personally meaningful or emotionally sensitive.

Third, the study focused on prototype development, refinement, and early usability evaluation. It did not assess the portal’s impact on behavioural outcomes, blood pressure control, recurrence risk, shared decision-making, healthcare use, or clinical outcomes. The findings should therefore be interpreted as preliminary evidence of acceptability, perceived usefulness, and usability, rather than evidence that the portal improves secondary prevention or recovery outcomes.

Fourth, although the portal was informed by prior qualitative and PPIE work, the present study primarily reports the translation of user-derived findings into a functional prototype and the formative evaluation of that prototype. Stroke survivors and carers contributed through prior qualitative/PPIE work, prototype feedback, and usability testing, but further work should involve users more directly in implementation planning, prioritisation of future features, and evaluation of real-world use.

Finally, despite the accessibility-oriented features incorporated into the prototype, independent use may still be affected by differences in digital literacy, cognitive or communication impairments, device access, and confidence with technology. Future work should therefore evaluate onboarding, training, device flexibility, and adaptive support features before wider implementation.

## Conclusions

This study presents the public involvement and engagement-informed user-centred development and early usability evaluation of a digital portal designed to support post-stroke self-management. Grounded within the South London Stroke Register, the prototype translated longitudinal stroke-related data into a patient-facing system integrating personal health records, home blood pressure monitoring, recurrence-risk information, self-assessment tools, educational resources, and caregiver contact information.

Usability testing with eight stroke survivors provided preliminary evidence of acceptability, perceived usefulness, and practical usability in a supported testing environment, and identified priorities for further refinement before feasibility evaluation. Overall, this study illustrates how prior user-derived evidence and multidisciplinary feedback can be translated into a functional stroke register-linked portal prototype. Future feasibility work should examine real-world acceptability, implementation, workflow integration, and use in a broader and more diverse stroke survivor population.

## Supplementary Information

Below is the link to the electronic supplementary material.

Supplementary file 1 (zip 112 KB)

## Data Availability

No datasets were generated or analysed during the current study.
