# Supplementary material for: PPIE-Informed User-Centred Development and Early Usability Evaluation of a Digital Patient Portal to Support Stroke Survivors’ Self-Management
Source: J Healthc Inform Res. 2026 Sep 17;10(4):922–51. doi: 10.1007/s41666-026-00253-9 (PMC13719948; doi:10.1007/s41666-026-00253-9)
Supplement: Supplementary file 1 [file 41666_2026_253_MOESM1_ESM.zip › Supplementary_Material_2.pdf]

Supplementary Material 2

Zhiqiang Huo[1,2], Timothy Neate[3], David Wyatt[2], Sophie Rowland-Coomber[2], Fara Hamidi[2], Martin Chapman[2], Yanzhong Wang[2], Iain J. Marshall[2], Charles D. A. Wolfe[2], Vasa Curcin[2]

[1]Wolfson Institute of Population Health, Queen Mary University of London, London, United Kingdom.

[2]Department of Population Health Sciences, King's College London, London, United Kingdom.

[3]Department of Informatics, King's College London, London, United Kingdom.

This supplementary material summarizes the user-derived requirements that informed the design, development and refinement of the stroke survivor patient portal.

Table S1. Functional requirements for supporting post-stroke self-management

Functional requirement	Underlying user or stakeholder need	Design implication for the portal
**Access to personalised stroke health information**	Stakeholders recognised the potential benefits of enabling stroke survivors and their families to access personalised health records. Stroke survivors wanted to better understand their recovery and observe positive progress after stroke. In the SLSR context, access to long-term follow-up data, stroke onset details, and medication history was considered important for informed care planning and tracking recovery.	Provide structured access to personalised stroke-related information, including stroke onset details, follow-up records, medication-related information, and longitudinal recovery information in a patient-facing format.
**Supporting stroke recovery through health monitoring**	Stroke survivors used both digital platforms, such as NHS and GP apps, and analogue tools, such as notebooks and calendars, to monitor their health at home. A stroke survivor noted that a blood pressure monitor was helpful because it stored multiple records and showed an average reading. Stakeholders highlighted the need for a centralised health tracking tool that could integrate different forms of health data.	Include home health monitoring functions, particularly blood pressure logging, trend visualisation, plain-language interpretation, step-by-step guidance for accurate self-assessment, and printable summaries that could support GP or clinical discussions.
**Health self-assessment tools**	Stakeholders recommended health self-assessment tools to support continuous monitoring and active participation in post-stroke recovery. Measures such as the Barthel Index, EQ-5D-5L, and Fatigue Severity Score were considered useful for helping survivors understand changes in physical function, quality of life, and energy levels over time. Participants also raised concerns that binary labels or strong warning cues could be discouraging.	Present self-assessment tools with supportive explanations, flexible visualisation, and careful wording. Avoid discouraging binary labels such as "recovered/not recovered" and avoid unnecessarily alarming visual cues. Allow recovery-related information to support awareness and collaborative care without reducing motivation.
**Centralised educational and support resources**	PPIE contributors highlighted the need for a centralised educational and local support hub. Many stroke survivors reported difficulty locating credible and relevant stroke information. Caregivers also emphasised the value of emergency or caregiver contact information to support coordinated care and communication after discharge.	Provide a searchable and centralised route to trusted educational content, local stroke recovery services, communication support resources, and personalised caregiver or emergency contact information.

Table S2. User experience and accessibility requirements informing prototype refinement

User experience or accessibility requirement	Rationale from prior qualitative/PPIE and stakeholder feedback	Design response or refinement
**Use plain and patient-friendly language**	Content clarity was a recurring requirement. Participants and contributors emphasised that clinical terminology should be translated into plain, everyday language to support users with different levels of health literacy.	Avoid presenting medication classes and clinical terms without explanation. Use plain-language descriptions such as “a medicine to lower blood pressure” or “a medicine to prevent blood clots” across personal health record, blood pressure, and educational resource sections.
**Clarify what is meant by personal health data**	Feedback indicated that the word “data” could be too broad or ambiguous. Stroke survivors and informal carers could interpret it as diagnostic details, follow-up records, medication history, blood pressure readings, or self-assessment scores.	Use specific labels for information categories, including stroke progress, health measurements, follow-up history, blood pressure readings, and educational resources. Add contextual explanations to clarify what information is being shown and why it is relevant to self-management.
**Provide trusted and centralised information**	Participants and contributors highlighted the need for reliable and easy-to-access information after stroke. Requiring users to search across multiple online sources could increase uncertainty and cognitive burden.	Include an educational resources section and centralised access to trusted stroke-related content and support resources, helping users locate relevant information without unnecessary searching.
**Present recovery trajectories and risk-related information sensitively**	Feedback highlighted the potential value of longitudinal health information, recovery trajectories, and risk-related visualisations, but also the need to avoid discouraging or distressing users if information was presented without supportive framing.	Use explanatory text, careful wording, and progressive disclosure. Present stroke recurrence risk in a way that emphasises modifiable factors and potential improvement rather than risk status alone.
**Reduce cognitive load and support accessible interaction**	Accessibility was central because stroke survivors may experience visual, cognitive, language, communication, or motor difficulties. Feedback supported a simple interface, clear navigation, consistent layouts, legible text, strong contrast, and minimal visual clutter.	Use a shallow navigation structure, grouped information panels, breadcrumb-style orientation cues, expandable sections, text-to-speech support, and close placement of blood pressure values, explanatory context, and interpretation.
**Support flexible use and future accessibility needs**	Users may vary in digital confidence, device preferences, and ability to type or interact with digital systems. Contributors highlighted the importance of future use across tablets and smartphones, as well as onboarding, training, and alternative input options.	Record device optimisation, onboarding, training materials, and alternative input options as future refinement priorities for feasibility evaluation and later implementation.
